# The effects of clonidine and yohimbine in the tail flick and hot plate tests in the naked mole rat (*Heterocephalus glaber*)

**DOI:** 10.1186/s13104-021-05607-7

**Published:** 2021-05-17

**Authors:** R. M. Mwobobia, T. I. Kanui, K. S. P. Abelson

**Affiliations:** 1 School of Agriculture and Veterinary Sciences, South Eastern Kenya University, P O Box 170-90200, Kitui, Kenya; 2 Department of Experimental Medicine, Faculty of Health and Medical Sciences, University of Copenhagen, Blegdamvej 3B, 2200 Copenhagen, Denmark

**Keywords:** Hot plate, Clonidine, Naked mole rats, Noradrenergic, Nociception, Tail flick, Yohimbine

## Abstract

**Objective:**

The naked mole rat (NMR) (*Heterocephalus glaber)* is increasingly considered an important biomedical research model for various conditions like hypoxic brain injury, cancer and nociception. This study was designed to investigate the effects of clonidine and yohimbine, an alpha-2 (*α*_2_) adrenoceptor agonist and antagonist respectively in the tail flick and hot plate tests.

**Results:**

A significant difference in tail flick latency was noted between saline control and 30 µg/kg clonidine, which was reduced after administration of 30 µg/kg yohimbine. A significant difference in hot plate latency was also noted between saline control and 30 µg/kg clodinine during the periods 30, 45, 60, 75 and 90 min after administration, and between saline control and 10 µg/kg clonidine during 30 min after administration. The hot plate latency by 30 µg/kg clonidine was also reduced by 30 µg/kg yohimbine during 30 min after administration. Since the tail-flick and hot plate tests mediate the effects at spinal and supraspinal levels respectively, the present study indicates the presence and involvement of noradrenergic receptors in thermal antinociception at spinal and supraspinal levels of the NMR, similar to what has been found in other mammals.

## Introduction

Nociception is the ability to detect painful stimuli like heat, chemical or mechanical energy [1]. NMRs are reportedly insensitive to itch induced by capsaicin, ammonia fumes, histamine and pain induced by acid [2–4]. Due to these unique characteristics, researchers have been bio prospecting NMRs as research animal models for nociception with the aim to further understand mechanisms involved in various biological diseases affecting humans [2, 3, 5].

The involvement of opioid [6, 7], cholinergic [8], 9 and GABA [10, 11] receptor pathways in NMR pain mechanisms has been reported. Noradrenergic receptor system involvement in nociception is reported in other animals such as; rats [12], pigs [13], dog and cats [14], Speke’s hinged tortoise [15], small ruminants [16], and frog [17]. To our knowledge, the involvement of the noradrenergic receptor system in antinociception against thermal stimuli has not been previously studied in the NMR.

The aim of the experiment was to study the role of the noradrenergic receptor system in the NMRs using the receptor agonist clonidine and antagonist yohimbine in the tail flick and hot plate tests, in order to establish whether NMR can be used as animal model for noradrenergic mechanisms in thermal antinociception. It was hypothesized that clonidine would reduce the tail flick and hot plate thermal induced nocifensive behaviors in a dose-related manner, and that this effect could be reversed or reduced by yohimbine.

## Main text

### Materials and methods

#### Animals, husbandry and study design

A total of 128 NMRs captured in Makueni County, Kenya, were kept at South Eastern Kenya University. Colonies composing of mixed sex were housed in Perspex cages with wood shavings as bedding material. The cages were partitioned into four equal chambers each measuring 35 × 25 × 20 cm in length, width and height respectively and joined by four tunnels, each measuring 30 × 5.5 × 5.5 cm in length, width and height respectively. Tunneling was to mimic underground burrows in their natural habitat.

Ventilation of the cages was achieved by covering them with plywood that was not closely fitting. Each captured colony was kept in its own cage. Specific age of the animals were unknown, but only adult animals we captured. During acclimatization and experiments, housing conditions were almost similar to those in the wild i.e. temperatures of 28- 31 °C and 24/0 dark/light cycle. Relative humidity was maintained at 50–70% to prevent drying and scaling of the NMR skin.

After one month of acclimatization, NMRs weighing between 25–35 g in groups of mixed sex per drug or control dose were used in the study with each animal acting as experimental unit. Each group of eight NMRs was injected with either clonidine (1, 3, 10 or 30 µg/kg), yohimbine (30 µg/kg), a combination of clonidine (30 µg/kg) and yohimbine (30 µg/kg), or saline (0.9% NaCl).

In order to avoid any bias related to possible sex differences, females and males were equally distributed in the groups. Each NMR was randomly selected and used only once. The experimenter was blinded and thus not aware of the drugs or vehicle injected until after data analysis. The sample size (n) was based on a power analysis resulting in a group size (n) of eight animals. A 50% decrease in mean latency for control animals was considered biologically relevant; the standard deviation value was estimated as 35% of the estimated treatment group mean value; the alpha was set to 0.05 and the power to 80%.

Tests were performed similar to [8] using tail flick and hot plate analgesiometers (H.L Scientific, Ambala, India). The hot plate test was performed using a hot plate analgesiometer (H.L Scientific, Ambala, India) which consisted of a copper plate (21 × 21 cm) enclosed in a 22.5 × 22.5 × 19 cm lidded perspex box. The NMRs were acclimatized to the cold plate for 30 min before start of the experiment. They were also observed to ascertain they had normal sensorimotor function during the acclimatization period by their ability to move within the box. Thirty minutes after receptor ligands and control injection, the NMRs were gently placed in the hot plate box preset at a fixed temperature of 60 ± 2 ºC and latency period between placement and start of escape behaviors was recorded. The cut off period was set at 30 s where animals that did not respond to the hot plate were withdrawn and assigned a response latency of 30 s.

For the tail flick test, NMR were acclimated to the restrainer for 30 min before start of the experiment. Thirty minutes after treatment, a radiant heat beam was focused on the midpoint of the tail until a flick of the tail. The latency period was then recorded in seconds. NMRs that reached the cutoff point of 10 s were withdrawn to avoid injury and assigned a response latency of 10 s.

Volumes of test ligands or controls injected were adjusted for the weight of each animal to ensure that each animal received the correct dose. Each animal was euthanized by cervical dislocation after the experiment. Both experiments were carried out during daytime, between 8.30 and 17.00 h at a room temperature range of 27.2–28.9 °C.

#### Drugs

Clonidine hydrochloride (CL) and yohimbine hydrochloride (Yoh) both from Sigma-Aldrich (Taufkirchen, Germany) and 0.9% normal saline (NS).

#### Statistical analysis

Data were analyzed in Graph Pad Prism version 5.0. *P* < 0.05 was considered a statistically significant difference.

## Results

**Fig. 1:**
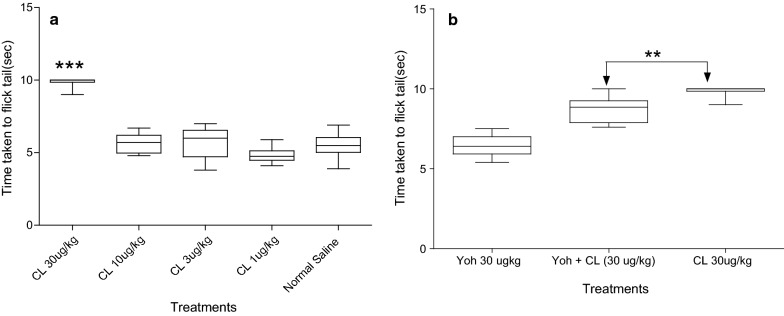
**a** The effects of CL at various doses (treatment) and NS (control). Significant differences (*p* < 0.0001***) in tail flick latency were found between 30 µg/kg CL compared to NS and CL dosages 1, 3 & 10 µg/kg. **b** Latency period when CL was co administered with Yoh significantly differed (*p* < 0.05**) with the effects of 30 µg/kg CL alone. The whiskers show minimum and maximum latency periods while the horizontal bar within the boxes show median latency in seconds. Number of animals (n) = 8 for all doses. Data were analyzed using one way ANOVA with Tukey's Multiple Comparison Test

**Fig. 2:**
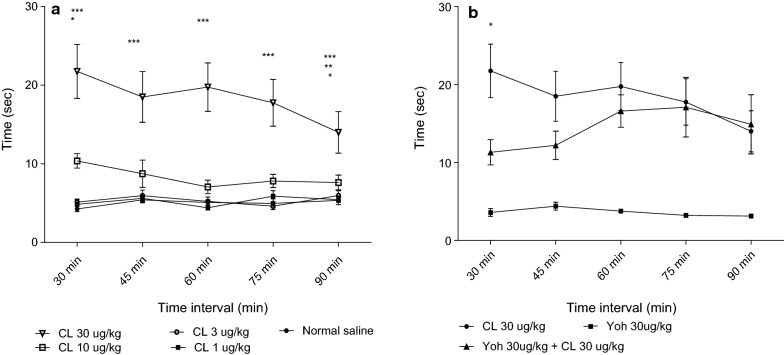
**a** Effect of NS (control) and CL (treatment) at various doses on hot plate latency. There were significant differences when; 30 µg/kg CL was compared to NS control and 1, 3, 10 µg/kg CL (*P* < 0.001***) at 30, 45, 60 and 75 min; NS and 10 µg/kg CL (*P* < 0.05*) when compared at 30 min; 30 µg/kg CL when compared to 10 µg/kg CL (P < 0.05*), 3 µg/kg CL (*P* < 0.01**) & NS (*P* < 0.001***) at 90 min **b** Latency period when Yoh was co administered with CL significantly differed with that of CL administered alone at 30 min (*P* < 0.05*). Data are shown as mean time (± SEM) in seconds. Number of animals (n) = 8 for all doses. Data were analyzed using Two way repeated measures ANOVA with Bonferroni posttests

Doses used in this study were based on studies by [18]. The effect of 1–30 µg/kg CL and NS control on tail flick latency is shown in Fig. 1a. The effect of 1–30 µg/kg CL and NS control on the hot plate latency is shown in Fig. 2a. The effects of co-administration of Yoh with CL in both the tail flick and hot plate tests is shown in Figs. 1b and 2b respectively.

## Discussion

Noradrenergic receptors play an inhibitory role at pre and post synaptic neurons and occur at both central (CNS) and peripheral nervous systems (PNS) where they function as G protein-coupled receptors [19, 20]. They are divided into α1 and α2 types [21, 22].

Activation of α2 adrenoceptors by their agonists like CL causes sedation, muscle relaxation and analgesia [23, 24]. CL has a high affinity for α2 adrenoceptors at both CNS and PNS with a predilection of 200:1 for α2 compared to α1 adrenoceptors [23]. Yoh is a prototypical α2 adrenergic receptor antagonist due to its high selectivity [25]. To study the spinal nociceptive reflexes, we performed the tail flick test where noxious thermal stimuli from radiant heat is applied on the tail [26]. The significant difference in tail flick when 30 µg/kg CL latency period was compared to that of NS and the reversal of the effects by Yoh indicates the presence and involvement of noradrenergic receptors at spinal level in the NMR. The increase in tail flick latency following administration of CL and the reversal of the effects by Yoh is also reported in rats [27] and mice [28, 29].

To study supra spinal effects, we performed the hot plate test where thermal noxious stimuli from a hot plate heated at 60 ± 2 ºC was applied [8, 30]. Significant difference in hot plate latency when 30 µg/kg CL was compared to that of NS and the reversal of the effects by Yoh indicates the presence and involvement of noradrenergic receptors at supra spinal level in the NMR. This is also reported in rats [31] and mice (28).

In conclusion, this study demonstrates involvement of noradrenergic receptor system in thermal antinociception of NMR at both CNS and PNS levels, similar to what has been reported in other rodents such as rats and mice. This suggests that the NMR can serve as an animal model for studying pain mechanisms involving the noradrenergic receptor system, as hypothesized. Future studies should aim to establish any interaction between noradrenergic system and the ligands used to other receptor systems in NMR as well as comparing the genome for noradrenergic receptors to other experimental rodents like mice and rats.

## Limitations

Wild type NMR of unknown ages were used, and their genetic profile was likely diverse, giving the experiments a lower precision. The ligands used may also have affected other receptor systems, hence the effects noted may not be purely due to noradrenergic receptors involvement.

## Data Availability

All the data and results generated and analyzed have been presented in this published article. Raw data, statistical calculations and power analysis input data are publicly available on Figshare.com (https://doi.org/10.6084/m9.figshare.14501379).

## Abbreviations

NMR: Naked mole rat
GABA: Gamma aminobutyric acid
α: Alpha
kg: Kilograms
µg: Micro grams
CNS: Central nervous system
PNS: Peripheral nervous system
NACL: Sodium chloride
